# For a Few Dollars More: Verified Fine-Grained Algorithm Analysis Down to LLVM

**DOI:** 10.1007/978-3-030-72019-3_11

**Published:** 2021-03-23

**Authors:** Maximilian P. L. Haslbeck, Peter Lammich

**Affiliations:** Imperial College, London, UK; 9 Technische Universität München, München, Germany; 10 The University of Manchester, Manchester, England

**Keywords:** Algorithm Analysis, Program Verification, Refinement

## Abstract

We present a framework to verify both, functional correctness and worst-case complexity of practically efficient algorithms. We implemented a stepwise refinement approach, using the novel concept of *resource currencies* to naturally structure the resource analysis along the refinement chain, and allow a fine-grained analysis of operation counts. Our framework targets the LLVM intermediate representation. We extend its semantics from earlier work with a cost model. As case study, we verify the correctness and $O(n\log n)$ worst-case complexity of an implementation of the introsort algorithm, whose performance is on par with the state-of-the-art implementation found in the GNU C++ Library.

### 1 Introduction

In general, not only correctness, but also the complexity of algorithms is important. While it is obvious that the performance *observed* during experiments is essential to solve practical problems efficiently, also the *theoretical* worst-case complexity of algorithms is crucial: a good worst-case complexity avoids timing regressions when hitting worst-case input, and, even more important, prevents denial of service attacks that intentionally produce worst-case scenarios to overload critical computing infrastructure.

For example, the C++ standard requires implementations of *std::sort* to have worst-case complexity $O(n \log n)$ [7]. Note that this rules out quicksort [12], which is very fast in practice, but has quadratic worst-case complexity. Nevertheless, some standard libraries, most prominently LLVM's *libc++* [20], still use sorting algorithms with quadratic worst-case complexity.[3]

A practically efficient sorting algorithm with $O(n \log n)$ worst-case complexity is Musser's introsort [22]. It combines quicksort with the $O(n \log n)$ heapsort algorithm, which is used as fallback when the quicksort recursion depth

[3] See, e.g., `https://bugs.llvm.org/show_bug.cgi?id=20837`.

exceeds a certain threshold. It allows to implement standard-compliant, practically efficient sorting algorithms. Introsort is implemented by, e.g., the GNU C++ Library (*libstdc++*) [8].

In this paper, we present techniques to formally verify both, correctness and worst-case complexity of practically efficient implementations. We build on two previous lines of research by the authors.

On one hand, we have the Isabelle Refinement Framework [19], which allows for a modular top-down verification approach. It utilizes stepwise refinement to separate the different aspects of an efficient implementation, such as algorithmic idea and low-level optimizations. It provides a nondeterminism monad to formalize programs and refinements, and the Sepref tool to automate canonical data refinement steps. Its recent LLVM back end [15] allows to verify algorithms with competitive performance compared to (unverified) highly optimized C/C++ implementations. The Refinement Framework has been used to verify the functional correctness of an implementation of introsort that performs on par with *libstdc++*'s implementation [17].

On the other hand, we already have extended the Refinement Framework to reason about complexity [11]. However, this only supports the Imperative/HOL back end [16]. It generates implementations in functional languages, which are inherently less efficient than highly optimized C/C++ implementations. This paper combines and extends these two approaches. Our main contributions are.

- We present a generalized nondeterminism monad with resource cost, apply it to resource functions to model fine-grained currencies (Section 2) and show how they can be used to naturally structure refinement.
- We extend the LLVM back end [15] with a cost model, and amend its basic reasoning infrastructure (Section 3).
- We extend the Sepref tool (Section 4) to synthesize executable imperative code in LLVM, together with a proof of correctness and complexity. Our approach seamlessly supports imperative and amortized data structures.
- We extend the verification of introsort to also show a worst-case complexity of $O(n \log n)$, thus meeting the C++11 *stdlib* specification [7] (Section 5). The performance of our implementation is still on par with *libstdc++*. We believe that this is the first time that both, correctness and complexity of a sorting algorithm have been formally verified down to a competitive implementation.

Our formalization is available at `https://www21.in.tum.de/~haslbema/llvm-time`.

### 2 Specification of Algorithms With Resources

We use the formalism of monads [24] to elegantly specify programs with resource usage. We first describe a framework that works for a very generic notion of *resource*, and then instantiate it with *resource functions*, which model resources of different *currencies*. We then describe a refinement calculus and show how currencies can be used to structure stepwise refinement proofs. Finally, we report on automation and give some examples.

#### 2.1 Nondeterministic Computations With Resources

Let us examine the features we require for our computation model.

First, we want to specify programs by their desired properties, without having to fix a concrete implementation. In general, those programs have more than one correct result for the same input. Consider, e.g., sorting a list of pairs of numbers by the first element. For the input $[(1,2),(2,2),(1,3)]$, both $[(1,2),(1,3),(2,2)]$ and $[(1,3),(1,2),(2,2)]$ are valid results. Formally, this is modelled as a *set* of possible results. When we later fix an implementation, the set of possible results may shrink. For example, the (stable) insertion sort algorithm always returns the list $[(1,2),(1,3),(2,2)]$. We say that insertion sort *refines* our specification of sorting.

Second, we want to define recursion by a standard fixed-point construction over a flat lattice. The bottom of this lattice must be a dedicated element, which we call `fail`. It represents a computation that may not terminate.

Finally, we want to model the resources required by a computation. For nondeterministic programs, these may vary depending on the nondeterministic choices made during the computation. As we model computations by their possible results, rather than by the exact path in the program that leads to the result, we also associate resource cost with possible results. When more than one computation path leads to the same result, we take the supremum of the used resources. The notion of refinement is now extended to a subset of results that are computed using less resources.

We now formalize the above intuition: The type

$$(\alpha,\gamma)\ \mathit{NREST} = \texttt{fail} \mid \texttt{res}\ (\alpha \to \gamma\ \mathit{option})$$

models a nondeterministic computation with results of type $\alpha$ and resources of type $\gamma$.[4] That is, a computation is either `fail`, or `res` $M$, where $M$ is a partial function from possible results to resources.

We define `spec` $\Phi\ T$ as a computation of any result $r$ that satisfies $\Phi\ r$ using $T\ r$ resources: `spec` $\Phi\ T$ = `res` $(\lambda r.$ `if` $\Phi\ r$ `then` $\mathit{Some}\ (T\ r)$ `else` $\mathit{None})$. By abuse of notation, we write `spec` $x\ T$ for `spec` $(\lambda r.\ r{=}x)\ (\lambda\_.\ T)$.

Based on an ordering on the resources $\gamma$, we define the *refinement ordering* on NREST, by first lifting the ordering to *option* with *None* as the bottom element, then pointwise to functions and finally to $(\alpha,\gamma)$ *NREST*, setting `fail` as the top element. This matches the intuition of refinement: $m \le m'$ reads as *$m$ refines $m'$*, i.e., $m$ has less possible results than $m'$, computed with less resources.

We require the resources $\gamma$ to have a complete lattice structure, such that we can form suprema over the (possibly infinitely many) paths that lead to the same result. Moreover, when sequentially composing computations, we need to add up the resources. This naturally leads to a monoid structure $(\gamma, 0, +)$, where 0, intuitively, stands for no resources.

We call such types $\gamma$ *resource types*, if they have a complete lattice and monoid structure. Note that, in an earlier iteration of this work [11], the resource type

[4] The name NREST abbreviates **N**ondeterministic **RES**ult with **T**ime, and has been inherited from our earlier formalizations.

was fixed to extended natural numbers (*enat*=$\mathbb{N} \cup \{\infty\}$), measuring the resource consumption with a single number. Also note that $(\alpha, unit)$ *NREST* is isomorphic to our original nondeterministic result monad without resources [19].

If $\gamma$ is a resource type, so is $\eta \to \gamma$. Intuitively, such resources consist of coins of different *resource currencies* $\eta$, the amount of coins being measured by $\gamma$.

*Example 1.* In the following we use the resource type $ecost = string \to enat$, i.e., we have currencies described by a string, whose amount is measured by extended natural numbers, where $\infty$ models arbitrary resource usage. Note that, while the resource type $string \to enat$ guides intuition, most of our theory works for general resource types of the form $\eta \to \gamma$ or even just $\gamma$.

We define the function $\$_s\ n$ to be the resource function that uses $n :: enat$ coins of the currency $s :: string$, and write $\$_s$ as shortcut for $\$_s\ 1$.

A program that sorts a list in $O(n^2)$ can be specified by:

$$sort_{spec}\ xs = \texttt{spec}\ (\lambda xs'.\ sorted\ xs' \wedge mset\ xs' = mset\ xs)\ (\$_q\ |xs|^2 + \$_c)$$

that is, a list $xs$ can result in any sorted list $xs'$ with the same elements, and the computation takes (at most) quadratically many $q$ coins in the list length, and one c coin, independently of the list length. Intuitively, the $q$ and $c$ coins represent the constant factors of an algorithm that implements that specification and are later elaborated by exchanging them into several coins of more fine-grained currencies, corresponding to the concrete operations in the algorithm, e.g., comparisons and memory accesses. Abstract currencies like $q$ and $c$ only *"have value"* if they can be exchanged to meaningful other currencies, and finally pay for the resource costs of a concrete implementation.

### 2.2 Atomic Operations and Control Flow

In order to conveniently model actual computations, we define some combinators. The `elapse` $m\ t$ combinator adds the (constant) resources $t$ to all results of $m$:

$$\begin{aligned}
&\texttt{elapse} :: (\alpha,\gamma)\ NREST \to \gamma \to (\alpha,\gamma)\ NREST\\
&\texttt{elapse fail}\ t = \texttt{fail}\\
&\texttt{elapse (res}\ M)\ t = \texttt{res}\ (\lambda x.\ \texttt{case}\ M\ x\ \texttt{of}\ None \Rightarrow None\\
&\qquad\qquad\qquad\qquad\qquad\qquad\quad |\ Some\ t' \Rightarrow Some\ (t + t'))
\end{aligned}$$

The program[5] `return` $x$ computes the single result $x$ without using any resources:

$$\begin{aligned}
&\texttt{return} :: \alpha \to (\alpha,\gamma)\ NREST\\
&\texttt{return}\ x = \texttt{res}\ [\ x \mapsto 0\ ]
\end{aligned}$$

The combinator `bind` $m\ f$ models the sequential composition of computations $m$ and $f$, where $f$ may depend on the result of $m$:

[5] Note that our shallow embedding makes no formal distinction between syntax and semantics. Nevertheless, we refer to an entity of type *NREST*, as *program* to emphasize the syntactic aspect, and as *computation* to emphasize the semantic aspect.

$$\texttt{bind} :: (\alpha,\gamma)\ \mathit{NREST} \to (\alpha \to (\beta,\gamma)\ \mathit{NREST}) \to (\beta,\gamma)\ \mathit{NREST}$$
$$\texttt{bind fail}\ f = \texttt{fail}$$
$$\texttt{bind}\ (\texttt{res}\ M)\ f = \mathit{Sup}\ \{\ \texttt{elapse}\ (f\ x)\ t\ |x\ t.\ M\ x = \mathit{Some}\ t\ \}$$

If the first computation $m$ fails, then also the sequential composition fails. Otherwise, we consider all possible results $x$ with resources $t$ of $m$, invoke $f\ x$, and add the cost $t$ for computing $x$ to the results of $f\ x$. The supremum aggregates the cases where $f$ yields the same result, via different intermediate results of $m$, and also makes the whole expression fail if one of the $f\ x$ fails.

*Example 2.* We now illustrate an effect that stems from our decision to aggregate the resource usage of different computation paths that lead to the same result. Consider the program

$$\texttt{res}\ (\lambda n{::}nat.\ \mathit{Some}\ (\$_c\ n));\ \texttt{return}\ 0$$

It first chooses an arbitrary natural number $n$ consuming $n$ coins of currency $c$, and then returns the result *0*. That is, there are arbitrarily many paths that lead to the result 0, consuming arbitrarily many $c$ coins. The supremum of this is $\infty$, such that the above program is equal to $\texttt{elapse}\ (\texttt{return}\ 0)\ (\$_c\ \infty)$. Note that none of the computation paths actually attains the aggregated resource usage. We will come back to this in Section 4.4.

Finally, we use Isabelle/HOL's *if-then-else* and define a recursion combinator `rec` via a fixed-point construction [13], to get a complete set of basic combinators. As these combinators also incur cost in the target LLVM, we define resource aware variants. Furthermore we also derive a while combinator:

$$\texttt{if}_c\ b\ \texttt{then}\ c_1\ \texttt{else}\ c_2 = \texttt{elapse}\ (r \leftarrow b;\ \texttt{if}\ r\ \texttt{then}\ c_1\ \texttt{else}\ c_2)\ \$_{if}$$
$$\texttt{rec}_c\ F\ x = \texttt{elapse}\ (\texttt{rec}\ (\lambda D\ x.\ F\ (\lambda x.\ \texttt{elapse}\ (D\ x)\ \$_{call})\ x)\ x)\ \$_{call}$$
$$\texttt{while}_c\ b\ f\ s = \texttt{rec}_c\ (\lambda D\ s.\ \texttt{if}_c\ b\ s\ \texttt{then}\ s \leftarrow f\ s;\ D\ s\ \texttt{else return}\ s)\ s$$

Here, the guard of $\texttt{if}_c$ is a computation itself, and we consume an additional $if$ coin to account for the conditional branching in the target model. Similarly, every recursive call consumes an additional *call* coin.

*Assertions* fail if their condition is not met, and return *unit* otherwise:

$$\texttt{assert}\ P = \texttt{if}\ P\ \texttt{then return}\ ()\ \texttt{else fail}$$

They are used to express preconditions of a program. A Hoare-triple for program $m$, with precondition $P$, postcondition $Q$ and resource usage $t$ is written as a refinement condition: $m \le \texttt{assert}\ P;\ \texttt{spec}\ Q\ (\lambda\_.\ t)$

*Example 3.* Comparison of two list elements at a cost of $t$ can be specified by:

$$\mathit{idxs\_cmp}_{spec}\ xs\ i\ j\ (t) = \texttt{assert}\ (i{<}|xs| \wedge j{<}|xs|);\ \texttt{spec}\ (xs!i < xs!j)\ (\lambda\_.\ t)$$

where $xs!i$ is the $i$th element of list $xs$. Instead of fixing the cost for specifications, we pass them as parameter $t$. This allows us to refine different instances of abstract data types (here lists) by different concrete data structures with different costs. To make bigger programs more readable, we note the cost parameter in parenthesis at the end of the line, as, e.g., in Example 4.

#### 2.3 Refinement on NREST

We have used the refinement ordering to express Hoare triples. Two other applications of refinement are data refinement and currency refinement.

*Data Refinement* A typical use-case of refinement is to implement an *abstract* data type by a *concrete* data type. For example, we could implement (finite) sets of numbers by sorted lists. We define a *refinement relation* $R$ between sorted lists and sets. A concrete computation $m_\dagger$ that yields sorted lists then refines an abstract computation $m$ that yields sets, if every possible concrete result is related to a possible abstract result. Formally, $m_\dagger \le \Downarrow_D R\ m$, where the operator $\Downarrow_D$ is defined, for arguments $R$ and $m$, by the following two rules.

$$\Downarrow_D R\ (\texttt{res}\ M) = \texttt{res}\ (\lambda c.\ Sup\ \{M\ a \mid a.\ (c,a) \in R\}) \qquad \Downarrow_D R\ \texttt{fail} = \texttt{fail}$$

Again, we use the supremum to aggregate the costs of all abstract results that are related to a concrete result. As in Example 2, this leads to the possibility that the supremum cost is not attained, which we discuss in Section 4.4.

*Currency Refinement* Consider we want to refine Example 3 into a program that first accesses the elements and then compares them.

*Example 4.* We refine $idxs\_cmp_{spec}$ $(\$_{idxs\_cmp})$ from Example 3 as follows:

$$\begin{array}{ll}
\multicolumn{2}{l}{idxs_cmp\ xs\ i\ j =} \\
\quad \texttt{assert}\ (i{<}|xs| \wedge j{<}|xs|); & \\
\quad xsi \leftarrow list_get_{spec}\ xs\ i; & (\$_{lookup}) \\
\quad xsj \leftarrow list_get_{spec}\ xs\ j; & (\$_{lookup}) \\
\quad \texttt{return}\ (xsi < xsj) & (\$_{less})
\end{array}$$

where $list\_get_{spec}\ xs\ i\ (T) = \texttt{assert}\ (i < |xs|);\ \texttt{spec}\ (xs!i)\ T$ and $\texttt{return}\ x\ (T)$ returns the result $x$ incurring cost $T$.

Note that $idxs\_cmp$ and $idxs\_cmp_{spec}$ use different, incompatible currency systems. To compare them, we need to exchange coins: one $idxs\_cmp$ coin will be traded for two *lookup* coins and one *less* coin.

To make that happen we introduce the currency refinement $\Downarrow_C E\ m$. Here, the *exchange rate* $E :: \eta_a \to \eta_c \to \gamma$ specifies for each abstract currency $c_a :: \eta_a$ how many of the coins of the concrete currency $c_c :: \eta_c$ are needed. Note that, in general, one abstract coin may be exchanged into multiple coins of different currencies. For a resource type $\gamma$ that provides a multiplication operation $(*)$ we define the operator $\Downarrow_C$ with the following two rules.

$$\begin{aligned}
\Downarrow_C E\ (\texttt{res}\ M) &= \texttt{res}\ (\lambda\ r.\ \texttt{case}\ M\ r\ \texttt{of}\ None \Rightarrow None\ | \\
&\qquad\qquad Some\ t \Rightarrow Some\ (\lambda c_c.\ \textstyle\sum_{c_a} t\ c_a * E\ c_a\ c_c)) \\
\Downarrow_C E\ \texttt{fail} &= \texttt{fail}
\end{aligned}$$

The refined computation has the same results as the original. To get the amount of a concrete coin $c_c$ for some result $r$ with resource function $t$, we sum, over all abstract coins $c_a$, the amount of abstract coins needed in the original computation ($t\ c_a$) weighted by the exchange rate ($E\ c_a\ c_c$).

For the sum to make sense, there must be only finitely many abstract coins $c_a$ with $t\ c_a * E\ c_a\ c_c \neq 0$. This can be ensured by restricting the resource functions $t$ of the computation to use finitely many different coins, or by restricting the exchange rate $E$ accordingly. The latter can be checked syntactically in practice.

*Example 5.* For refining the specification $idxs\_cmp_{spec}$ we can use the exchange rate $E_1 = 0(idxs\_cmp := \$_{lookup}\ 2 + \$_{less})$, which does the correct exchange for *idxs_cmp* and is zero everywhere else. Here, + and 0 are lifted to functions in a pointwise manner, and $f(\cdot := \cdot)$ denotes a function update. We can now prove:

$$idxs\_cmp\ xs\ i\ j \leq \Downarrow_C E_1\ (idxs\_cmp_{spec}\ xs\ i\ j\ (\$_{idxs\_cmp}))$$

#### 2.4 Refinement Patterns

In practice, we encounter certain recurring patterns of refinement, which we describe in this section.

*Refinement of Specifications* Instead of only asking *whether* a program $m$ satisfies a specification `res` $M$, we also ask *how much* it satisfies the specification, i.e. what is the difference of the resources specified and actually used, denoted by $gwp\ m\ M$.[6] We have the following equality: $m \leq \texttt{res}\ M \Leftrightarrow Some\ 0 \leq gwp\ m\ M$.

To get some intuition let us fix the resource to be time. Then, $gwp\ m\ M$ is the *latest* feasible time at which we can start $m$ to still match the deadline $M$. If there is no feasible starting time ($gwp\ m\ M = None$), $m$ does not fulfill the specification $M$. If it has some value $t$, this is the latest feasible starting time of all computation paths in $m$.

Using *gwp*, we can implement a syntax driven verification condition generator, as already described in [11].

*Lockstep Refinement* We often refine a compound program by refining some of its components. Let $A$ and $C$ be two structurally equal programs (i.e., they have the same structure of combinators $\texttt{if}_c$, $\texttt{rec}_c$, `bind`, etc.), and let $A_i$ and $C_i$ be the pairs of corresponding basic components, for $i \in \{0, \ldots, n\}$. Provided with refinement lemmas $\Phi_i\ x \wedge (x_\dagger, x) \in R'_i \implies C_i\ x_\dagger \leq \Downarrow_D R_i\ (\Downarrow_C E\ (A_i\ x))$ for each of those pairs,[7] an automatic procedure walks through the program and establishes a refinement $C \leq \Downarrow_D R_n\ (\Downarrow_C E\ A)$. This process generates verification conditions for ensuring the preconditions $\Phi_i$, which can be discharged automatically or, if required, via interactive proof.

6 The definition of *gwp* requires $\gamma$ to provide a difference operator, dual to its + operator. It is a straightforward generalization of the concept defined in [11], and thus omitted here. We only note that the resource types *unit*, *enat*, and *ecost* provide a suitable difference operator.

7 The refinement relations $R'_i$ and $R_i$ relate the parameters and respectively the result of those components.

Note that, while the data refinements $R_i$ can be different for each component $i$, the exchange rate $E$ must be the same for all components. Currently, we align the exchange rates by manually deriving specialized versions of the component refinement lemmas. However, we believe that this can be automated in many practical cases, by collecting constraints on the exchange rate during the lockstep refinement, which are solved afterwards to obtain a unified exchange rate. We leave the implementation of this idea to future work.

*Separating Analysis of Resource Usage and Correctness* We can disregard resource usage and only focus on refinement of functional correctness, and then add resource usage analysis later. This is useful to separate the concerns of functional correctness and resource usage proof. We will describe a practical example later (Section 5.5), and only present an alternative way to prove the refinement in Example 4 here:

First, for functional correctness, we use the specification $idxs\_cmp_{spec}\ (\infty)$ and a program $idxs\_cmp_{\infty}$ similar to $idxs\_cmp$ but with all the costs replaced by $\infty$. Proving the refinement $idxs\_cmp_{\infty}\ xs\ i\ j \leq idxs\_cmp_{spec}\ xs\ i\ j\ (\infty)$ only requires showing verification conditions that correspond to functional properties and termination. In particular, assertions and annotated invariants in the concrete program have to be proved. Proof obligations on resource usage, however, collapse into the trivial $t \leq \infty$. For the same reason, we get $idxs\_cmp\ xs\ i\ j \leq idxs\_cmp_{\infty}\ xs\ i\ j$, and by transitivity obtain

$$idxs\_cmp\ xs\ i\ j \leq idxs\_cmp_{spec}\ xs\ i\ j\ (\infty)$$

Next, we prove $idxs\_cmp\ xs\ i\ j \leq_n \mathtt{spec}\ (\lambda\_.\ True)\ (\$_{lookup}\ 2 + \$_{less})$. Here, the refinement relation $m \leq_n m' = m \neq \mathtt{fail} \implies m \leq m'$ assumes that the concrete program does *not* fail. This has the effect that, during the refinement proof, assertions and annotated invariants in the concrete program can be assumed to hold, and we can focus on the resource usage proof.

Finally, the two refinements can be combined to obtain

$$idxs\_cmp\ xs\ i\ j \leq idxs\_cmp_{spec}\ xs\ i\ j\ (\$_{lookup}\ 2 + \$_{less})$$

## 3 LLVM With Cost Semantics

The NREST-monad allows to specify programs with their resource usage in abstract currencies. Those currencies only have a meaning when they finally can be exchanged for the costs of concrete computations. In the following we present such a concrete computation model, namely a shallow embedding of the LLVM semantics into Isabelle/HOL. The embedding is an extension of our earlier work [15] to also account for costs. In Section 4 we then report on linking the LLVM back end with the NREST front end.

### 3.1 Basic Monad

At the basis of our LLVM formalization is a monad that provides the notions of non-termination, failure, state, and execution costs.

$\alpha\ mres = NTERM \mid FAIL \mid SUCC\ \alpha\ cost\ state$
$\alpha\ M = state \to \alpha\ mres$

Here, *cost* is a type for execution costs, which forms a monoid with operation + and neutral element 0, and *state* is an arbitrary type.[8]

The type $\alpha\ M$ describes a program that, when executed on a state, either does not terminate (*NTERM*), fails (*FAIL*), or returns a result of type $\alpha$, its execution costs, and a new state (*SUCC*).

It is straightforward to define the monad operations `return` and `bind`, as well as a recursion combinator `rec` over *M*. Thanks to the shallow embedding, we can also use Isabelle HOL's *if-then-else* to get a complete set of basic operations. As an example, we show the definition of the `bind` operation, in the case that both arguments successfully compute a result:

Assume $m\ s = SUCC\ x\ c_1\ s_1$ and $f\ x\ s_1 = SUCC\ r\ c_2\ s_2$
then we have `bind` $m\ f\ s = SUCC\ r\ (c_1{+}c_2)\ s_2$

That is, the result $x$ and state $s_1$ after the first operation $m$ is passed into the second operation *f*, and the result and state after the `bind` is what emerges from *f*. The cost for the `bind` is the sum of the costs for both operations.

The basic monad operations do not cost anything. To account for execution costs, we define an explicit operation `consume` $c\ s = SUCC\ ()\ c\ s$.[9]

#### 3.2 Shallowly Embedded LLVM Semantics

The formalization of the LLVM semantics is organized in layers. At the bottom, there is a memory model that stores deeply embedded values, and comes with basic operations for allocation/deallocation, loading, storing, and pointer manipulation. Also the basic arithmetic operations are defined on deeply embedded integers. These operations are phrased in the basic monad, but consume no costs. This way, we could take them unchanged from our original LLVM formalization without cost [15]. For example, the low-level load operation has the signature *raw_load :: raw_ptr → val M*. Here, *raw_ptr* is the pointer type of our memory model, consisting of a block address and an offset, and *val* is our value type, which can be an integer, a pointer, or a pair of values.

On top of the basic layer, we define operations that correspond to the actual LLVM instructions. Here, we map from deeply embedded values to shallowly embedded values, and add the execution costs.

For example, the semantics of LLVM's load instruction is defined as follows:

[8] Note that this differs from the NREST monad in Section 2.1: it is deterministic, and provides a state. Because of determinism, we never need to form a supremum, and thus can base our cost model on natural numbers rather than enats. We leave a unification of the two monads to future work.

[9] For NREST, we defined a *higher-order* operation `elapse`, while we use the *first-order* operation `consume` here. This is for historical reasons. Note that `elapse` can be defined in terms of `consume`, and vice versa.

$$
\begin{array}{l}
\mathit{ll\_load} :: \alpha\ \mathit{ptr} \to \alpha\ M \\
\mathit{ll\_load}\ p = \\
\quad \texttt{consume}\ \$_{load}; \\
\quad r \leftarrow \mathit{raw\_load}\ (\mathit{the\_raw\_ptr}\ p); \\
\quad \mathit{checked\_from\_val}\ r
\end{array}
$$

It consumes the cost[10] for the operation, and then forwards to the *raw_load* operation of the lower layer, where *the_raw_ptr* and *checked_from_val* convert between the shallow and deep embedding of values.

Like in the original formalization[11], an LLVM program is represented by a set of monomorphic constant definitions of the shape *def*, defined as follows:

$$
\begin{array}{l}
\mathit{def} = \mathit{proc\_name}\ \mathit{var}^{*} \equiv \mathit{block} \\
\mathit{block} = \mathit{var} \leftarrow \mathit{cmd};\ \mathit{block} \mid \texttt{return}\ \mathit{var} \\
\mathit{cmd} = \mathit{ll\_{<}opcode{>}}\ \mathit{arg}^{*} \mid \mathit{ll\_call}\ \mathit{proc\_name}\ \mathit{arg}^{*} \mid \mathit{llc\_if}\ \mathit{arg}\ \mathit{block}\ \mathit{block} \\
\qquad \mid \mathit{llc\_while}\ \mathit{block}\ \mathit{block} \\
\mathit{arg} = \mathit{var} \mid \mathit{number} \mid \mathit{null} \mid \mathit{init}
\end{array}
$$

The code generator checks that the set of definitions is complete and adheres to the required shape. It then translates them into LLVM code, which merely amounts to pretty printing and translating the structured control flow by `if` and `while`[12] statements to the unstructured control flow of LLVM. A powerful preprocessor can convert a more general class of terms to the restricted shape required by the code generator. This conversion is done inside the logic, i.e., the processed program is proved to be equal to the original. Preprocessing steps include monomorphization of polymorphic constants, extraction of fixed-point combinators to recursive function definitions, and conversion of tuple constructors and destructors to LLVM's *insertvalue* and *extractvalue* instructions.

In summary, the layered architecture of our LLVM formalization allowed for a smooth integration of the cost aspect, reusing most of the existing formalization nearly unchanged. Note that we opted to integrate the cost aspect into the existing top layer, which converts between deep and shallow embedding. Alternatively, we could have added another layer on top of the shallow embedding. While the latter would have been the cleaner design, we opted for the former approach to avoid the boilerplate of adding a new layer. This was feasible as the original top layer was quite thin, such that adding another aspect there did not result in excessive complexity.

[10] See Section 3.3 for an explanation of our cost model.

[11] Actually, the only change to the original formalization is the introduction of the *ll_call* instruction, to make the costs of a function call visible.

[12] Primitive while loops are not strictly required, as they can always be replaced by tail recursion. Indeed, our code generator can be configured to not accept while loops, and our preprocessor can automatically convert while loops to tail-recursive functions. However, the efficiency of the generated code then relies on LLVM's optimization pass to detect the tail recursion and transform it to a loop again.

#### 3.3 Cost Model

As a cost model for running time, we chose to count how often each instruction is executed. That is, we set $cost = string \rightarrow nat$, where the string encodes the name of an instruction. It is straightforward to define $0$ and $+$ such that $(cost, 0, +)$ forms a monoid. It is thus a valid cost model for our monad.

But how realistic is our cost model, counting LLVM instructions? During compilation, LLVM text will be transformed by LLVM's optimizer, and finally, the LLVM's back end will translate LLVM instructions to machine instructions. Moreover, the actual running time of a machine program does not only depend on the number of executed instructions, but effects like pipeline flushes and cache misses also play an important role. Thus, without factoring in the details of the optimization passes and the target machine architecture, our cost model can, at best, be a rough approximation of the actual running time.

However, we can sensibly assume that a single instruction in the original LLVM text will result in at most a (small) constant number of machine instructions, and that each machine instruction has a constant worst-case execution time. Thus, the steps counted by our model linearly correlate to an upper bound of the actual execution time, though the exact correlation depends on the actual program, optimizer passes, and target architecture. Hence, while our cost model cannot be used for precise statements about execution time, it can be used to prove worst-case complexity. That is, a program that we have proved efficient will be compiled to an efficient machine program. Moreover, we can hope that the constant factors in the proved complexity are related to the actual constant factors in the machine program, i.e., an LLVM program with small constant factors will compile to a machine program with small constant factors.

The above discussion justifies the following design choices: The *insertvalue* and *extractvalue* instructions, which are used to construct and destruct tuple values, have no associated costs. The main reason for this design is to enable transparent use of tupled values, e.g., to encode the state of a while loop. We expect LLVM to translate the members of the tuple to separate registers anyway, such that no real costs are associated with tupling/untupling.

We define the *malloc* instruction to take cost proportional to the number of allocated elements. Note that LLVM itself does not provide memory management, and our code generator forwards memory management instructions to the *libc* implementation of the target platform. We use the *calloc* function here, which is supposed to initialize the allocated memory with zeros. While the exact costs of that are implementation dependent, they certainly will depend on the size of the allocated block.

Charguéraud and Pottier [6, §2.7] discuss the adequacy of abstract cost models in a functional setting. In their classification, our abstraction is on Level 2.

#### 3.4 Reasoning Setup

Once we have defined the semantics, we need to set up some basic reasoning infrastructure. The original Isabelle-LLVM already comes with a quite generic separation logic and verification condition generation framework. Here, we report on our extensions to resources using time credits.

*Separation Logic with Time Credits* Our reasoning infrastructure is based on separation logic with time credits [1,6,10]. We follow the algebraic approach of Calcagno *et al.* [3], using an earlier extension [15] of Klein *et al.* [18].

A separation algebra on type $\alpha$ induces a *separation logic* on assertions that are predicates over $\alpha$. To guide intuition, elements of $\alpha$ are called *heaps* here. We use the following separation logic operators: The assertion $\uparrow\Phi$ holds for an empty heap if $\Phi$ holds, $\square{=}\uparrow True$ describes the empty heap, and $\exists_A$ is the existential quantifier lifted to assertions. The *separating conjunction* $P \star Q$ describes a heap comprised from two disjoint parts, one described by $P$ and the other described by $Q$, and entailment $P \vdash Q$ states that $Q$ holds for every heap described by $P$.

Separation algebras naturally extend over product and function types, i.e., for separation algebras $\alpha$, $\beta$, and any type $\gamma$, also $\alpha \times \beta$ and $\gamma \to \alpha$ are separation algebras, where the operations are lifted pointwise.

Note that *enat* forms a separation algebra, where elements, i.e. time credits, are always disjoint. Hence, also $ecost = string \to enat$, and $amemory \times ecost$ are separation algebras, where *amemory* is the separation algebra that we already used in [15] to describe the abstract memory of LLVM. Thus, $amemory \times ecost$ induces a separation logic with time credits that match our cost model. The *time credit assertion* $\$\,c = (\lambda a.\ a{=}(0{,}c))$ describes an empty memory ($0$) and precisely the time $c$. The primitive assertions on *amemory* are lifted analogously to describe no time credits.

*Weakest Precondition and Hoare Triples* We start by defining a concrete state *cstate* that describes the memory content and the available resources:

$$cstate = memory \times ecost$$

where *memory* is the memory type from our original LLVM formalization. Based on this, we define the weakest precondition predicate:

$$wp :: \alpha\ M \to (\alpha \to cstate \to bool) \to cstate \to bool$$
$$wp\ m\ Q\ (s{,}cc) = (\exists r\ c\ s'.\ m\ s = SUCC\ r\ c\ s' \wedge c{\le}cc \wedge Q\ r\ (s',\ cc{-}c)).$$

Intuitively, the costs *cc* stored in the state is the *credit* available to the program. The weakest precondition holds if the program runs with real costs $c$ that are within the available credit, and $Q$ holds for the result $r$, the new memory $s'$, and the new credit, $cc{-}c$, which is the old credit reduced by the actually required costs. Note that actual costs have type $cost = string \to nat$, i.e., are always finite, while the credits have type $ecost = string \to enat$, i.e., there can be infinite credits. Setting the credit to be infinite for all instruction types yields the classical weakest precondition that requires termination, but enforces no time limit.

Our concrete state type, in particular the memory, does not form a separation algebra, as the natural memory model of LLVM has no natural notion of partial memories. Thus, we define an abstraction function that maps a concrete state to an abstract state *astate*, which forms a separation algebra:

$$astate = amemory \times ecost \qquad\qquad abs\ (m,\ c) = (abs_m\ m,\ c)$$

Again, *amemory* and $abs_m$ is the abstract state and abstraction function from the original LLVM formalization. The costs already form a separation algebra, so we do not abstract them further.

With this, we can instantiate a generic VCG infrastructure: let *cstate* be concrete states, $wp :: \alpha\ M \to (\alpha \to cstate \to bool) \to cstate \to bool$ be a weakest precondition predicate, and *astate* an abstract state, linked to concrete states via an abstraction function $abs :: cstate \to astate$. Further, assume that *wp* distributes over conjunctions, i.e.,

$$wp\ c\ Q_1\ s \wedge wp\ c\ Q_2\ s \implies wp\ c\ (\lambda r\ s'.\ Q_1\ r\ s' \wedge Q_2\ r\ s')\ s$$

Finally, let $\mathbb{T}$ be an *affine top* [5], i.e., an assertion with $\Box \vdash \mathbb{T}$ and $\mathbb{T} \star \mathbb{T} = \mathbb{T}$, which captures resources that can be safely discarded. We define the *Hoare triple* $\{P\}\ c\ \{Q\}$ to hold iff:

$$\forall F\ s.\ (P{\star}F)\ (abs\ s) \implies wp\ c\ (\lambda r\ s'.\ (Q\ r \star \mathbb{T} \star F)\ (abs\ s'))\ s$$

Intuitively, $\{P\}\ c\ \{Q\}$ holds if, for all states that contain a part described by assertion $P$, command $c$ terminates with result $r$ and a state where that part is replaced by a part described by $Q\ r \star \mathbb{T}$, and the rest of the state has not changed. Here, $Q\ r$ is the postcondition of the Hoare triple, and $\mathbb{T}$ describes resources that may be left over and can be discarded.

In our case, we set $\mathbb{T}$ to describe the empty memory and any amount of time credits. This matches the intuition that a program must free all its memory, but may run faster than estimated, i.e., leave over some time credits. Note that our *wp* distributes over conjunctions.

The generic VCG infrastructure now provides us with a syntax driven VCG with a simple frame inference heuristics.

#### 3.5 Primitive Setup

Once we have defined the basic reasoning infrastructure, we have to prove Hoare triples for the basic LLVM instructions and control flow combinators. As we have added the cost aspect only at the top level of our semantics, we can reuse most of the material from our original LLVM formalization without time. Technically, we instantiate our reasoning infrastructure with a weakest precondition predicate *wpn*, which only holds for programs that consume no costs. We define:

$$wpn\ m\ Q\ s = wp\ m\ (FST \circ Q)\ (s,0)\ \textbf{where}\ FST\ P = \lambda(s,c).\ P\ s \wedge c{=}0$$

The resulting reasoning infrastructure is identical with the one of our original formalization, most of which could be reused. Only for the topmost level, i.e., for those functions that correspond to the functional semantics of the actual LLVM instructions, we lift the Hoare triples over *wpn* to Hoare triples over *wp*:

$$\{P\}\ c\ \{Q\}_{wpn} = \{FST\ P\}\ c\ \{FST \circ Q\}$$

*Example 6.* Recall the low-level *raw_load* and the high-level *ll_load* instruction from Section 3.2. The *raw_load* instruction consumes no costs, and our original LLVM formalization provides the following Hoare triple:

$$\{raw\_pto\ p\ x\}\ raw\_load\ p\ \{\lambda r.\ \uparrow(r{=}x) \star raw\_pto\ p\ x\}_{wpn}$$

This can be transferred to a Hoare triple over *wp*:

$$\{FST\ (raw\_pto\ p\ x)\}\ raw\_load\ p\ \{\lambda r.\ \uparrow(r{=}x) \star FST\ (raw\_pto\ p\ x)\}$$

which is then used to prove the Hoare triple for the program *ll_load*

$$\{\$\ \$_{load} \star pto\ p\ x\}\ ll\_load\ p\ \{\lambda r.\ \uparrow(r{=}x) \star pto\ p\ x\}$$

where $pto\ p\ x = FST\ (raw\_pto\ (the\_raw\_ptr\ p)\ (to\_val\ x))$.

Using the VCG and the Hoare triples for the LLVM instructions, we can now define and prove correct data structures and algorithms. While this works smoothly for simple data structures like arrays, it does not scale to more complex developments. In contrast, NREST *does* scale, but lacks support for the low-level pointer reasoning required for basic data structures. In the next section, we show how to combine both approaches, with the LLVM level providing basic data structures and the NREST level using them as building blocks for larger algorithms.

## 4 Automatic Refinement

In this section we describe a tool to synthesize a concrete program in the LLVM-monad from an abstract algorithm in the NREST-monad. It can automatically refine abstract functional data structures to imperative heap-based ones. We will describe the synthesis predicate *hnr* that connects the two monads, the synthesis tool, and a way to extract Hoare triples from *hnr* predicates. Finally, we will discuss an effect that prevents combining *hnr* with data refinements in the NREST-monad in the general case.

### 4.1 Heap nondeterminism refinement

The *heap nondeterminism refinement* predicate $hnr\ \Gamma\ m_\dagger\ \Gamma'\ R\ m$ intuitively expresses that the concrete program $m_\dagger$ computes a concrete result that relates, via the *refinement assertion* $R$, to a result in the abstract program $m$, using at most the resources specified by $m$ for that result. A refinement assertion describes how an abstract variable is refined by a concrete value on the heap. It can also contain time credits. The assertions $\Gamma$ and $\Gamma'$ constitute the heaps before and after the computation and typically are a separating conjunction of refinement assertions for the respective parameters of $m_\dagger$ and $m$. Formally, we define:

$$\begin{aligned}
&hnr\ \Gamma\ m_\dagger\ \Gamma'\ R\ m = m \neq \mathtt{fail} \implies \\
&\quad (\forall F\ s\ c.\ (\Gamma \star F)\ (abs_m\ s{,}c) \implies \\
&\qquad (\exists r_a\ c_a.\ \mathtt{elapse}\ (\mathtt{return}\ r_a)\ c_a \leq m \\
&\qquad\quad \wedge\ wp\ m_\dagger\ (\lambda r\ (s'{,}c').\ (\Gamma' \star R\ r\ r_a \star F \star \top)\ (abs_m\ s'{,}c'))\ (s,\ c{+}c_a)))
\end{aligned}$$

The predicate holds if either the abstract program fails or if, for all heaps and resources $(s,c)$ that satisfy the pre-assertion $\Gamma$ with some frame $F$, there exists an abstract result and cost $(r_a,c_a)$ that refine $m$, and $m_\dagger$ terminates with concrete result $r$ in a state $s'$ where $\Gamma'$ with the frame holds, and $r$ relates to the abstract result via assertion $R$. The execution costs of $m_\dagger$ and the time credits $c'$ required by the post-assertion $\Gamma'$ are paid for by the specified cost $c_a$ and the time credits $c$ described by the pre-assertion $\Gamma$. Thus, the real costs are paid by a combination of the advertised costs in the abstract program and the potential difference of $\Gamma'$ and $\Gamma$, allowing to seamlessly model amortized computation costs.

Using the affine top $\top$, it is possible for the program to throw away portions of the heap. Note that our $\top$ can only discard time credits. Memory must be explicitly freed by the concrete program $m_\dagger$.

Also note that *hnr* is not tied to the LLVM semantics specifically. It actually is a general pattern for combining the NREST-monad with any other program semantics that provides a weakest precondition and a separation algebra for data and resources.

#### 4.2 The Sepref Tool

The Sepref tool [14,15] automatically synthesizes a concrete program in the LLVM-monad from an abstract algorithm in the NREST-monad. It symbolically executes the abstract program while maintaining refinements for the abstract variables to a concrete representation and generates a concrete program as well as a valid *hnr* predicate. Proof obligations[13] that occur during this process are discharged automatically, guided by user-provided hints where necessary.

The synthesis requires rules for all abstract combinators. For example, `bind` is processed by the following rule:

1 $\llbracket\ \mathit{hnr}\ \Gamma\ m_\dagger\ \Gamma'\ R_x\ m;$
2 $\quad (\forall x\ x_\dagger.\ \mathit{hnr}\ (R_x\ x_\dagger\ x \star \Gamma')\ (f_\dagger\ x_\dagger)\ (R'_x\ x_\dagger\ x \star \Gamma'')\ R_y\ (f\ x));$
3 $\quad \mathit{MK\_FREE}\ R'_x\ \mathit{free}\ \rrbracket \implies$
4 $\mathit{hnr}\ \Gamma\ (x_\dagger \leftarrow m_\dagger;\ r_\dagger \leftarrow f_\dagger\ x_\dagger;\ \mathit{free}\ x_\dagger;\ \texttt{return}\ r_\dagger)\ \Gamma''\ R_y\ (x \leftarrow m;\ f\ x)$

To refine $x \leftarrow m;\ f\ x$, we first execute $m$, synthesizing the concrete program $m_\dagger$ (line 1). The state after $m$ is $R_x\ x_\dagger\ x \star \Gamma'$, where $x$ is the result created by $m$. From this state, we execute $f\ x$ and synthesize $f_\dagger\ x_\dagger$ (line 2). The new state is $R'_x\ x_\dagger\ x \star \Gamma'' \star R_y\ y_\dagger\ y$, where $y$ is the result of $f\ x$. Now, the intermediate variable $x$ goes out of scope and has to be deallocated. The predicate $\mathit{MK\_FREE}\ R'_x\ \mathit{free}$ (line 3) states that *free* is a deallocator for data structures implemented by refinement assertion $R'_x$. Note that *free* can only use time credits that are stored in $R'_x$. Typically, these are payed for during creation of the data structure. This way amortization can be used effectively to hide the necessary *free* operation and its costs in the abstract program.

All other combinators ($\texttt{rec}_c$, $\texttt{if}_c$, $\texttt{while}_c$, etc.) have similar rules that are used to decompose an abstract program into parts, synthesize corresponding con-

[13] E.g. from implementing mathematical integers with fixed-bit machine words.

crete parts recursively and combine them afterwards with the respective combinators from LLVM. At the leaves of this decomposition, atomic operations need to be provided with suitable synthesis predicates.

An example is a list lookup that is implemented by an array:

$$\begin{aligned}&hnr\ (array_A\ p\ xs \star snat_A\ i_\dagger\ i)\\&\quad(array\_nth\ p\ i_\dagger)\\&\ (array_A\ p\ xs \star snat_A\ i_\dagger\ i)\ id_A\ (list\_get_{spec}\ xs\ i\ (\lambda\_.\ array\_get_{cost}))\end{aligned}$$

where $array_A$, $snat_A$ and $id_A$ relate a list with an array, an unbounded natural number with a bounded signed word and identical elements respectively. With an array at address $p$ holding the list $xs$ and an index $i_\dagger$ that is a bounded signed word representing an unbounded natural number $i$, $array\_nth$ leaves the parameters unchanged and extracts the element specified by $list\_get_{spec}$ incurring costs $array\_get_{cost}$=$\$_{ofs\_ptr} + \$_{load}$.

Ideally, each operation has its own currency (e.g. $list\_get$). However, as our definition of $hnr$ does not support currency refinement, the basic operations must use the currencies of the LLVM cost model. To still obtain modular $hnr$ rules, we encapsulate specifications for data structures with their cost, e.g. by defining $array\_get_{spec}$=$list\_get_{spec}\ (\lambda\_.\ array\_get_{cost})$. These can easily be introduced in an additional refinement step. Automating this process, and possibly integrating currency refinement into $hnr$ is left to future work.

#### 4.3 Extracting Hoare Triples

Note that $hnr$ predicates cannot always be expressed as Hoare triples, as the running time bound of the abstract program may depend on the result, which we cannot refer to in the precondition of a Hoare triple, where we have to express the allowed running time as time credits. However, if the running time bound does not depend on the result, we can write $hnr$ as a Hoare triple:

$$hnr\ \Gamma\ m_\dagger\ \Gamma'\ R\ (\mathtt{spec}\ \Phi\ (\lambda\_.T)) = \{\$T{\star}\Gamma\}m_\dagger\{\lambda r.\ \Gamma'{\star}\exists_A r_a.\ R\ r\ r_a \star \uparrow(\Phi\ r_a)\}$$

While intermediate components might not be of this form, final algorithms typically are. At the end of a development, this rule allows to extract a Hoare triple in the underlying LLVM semantics, cutting out the NREST-monad. For validating the correctness claim of an algorithm, only the final Hoare triple needs to be inspected, which only uses concepts of the underlying semantics.

Note that the above rule is an equivalence. Thus, it can also be used to obtain synthesis rules from Hoare triples provided by the basic VCG infrastructure.

#### 4.4 Attain Supremum

We comment on a problem that arises when composing $hnr$ predicates and data refinement in the NREST monad. Consider the following programs and relations:

$$\begin{aligned}&m' = \mathtt{res}\ [x \mapsto \$_a,\ y \mapsto \$_b] &&\mathrm{R}_R = \{(\mathrm{z,a}),(\mathrm{z,b})\}\\&m = \mathtt{res}\ [z \mapsto \$_a + \$_b] &&\mathrm{R}_A = \mathrm{id}_A\\&m_\dagger = \mathtt{consume}\ (\$_a + \$_b);\ \mathtt{return}\ z\end{aligned}$$

Data refinement defines the resource bound for a concrete result (here $z$) as the supremum over all bounds of related results (here $x$, $y$). Thus, we have $m \leq \Downarrow_C R_R\ m'$. Moreover, we trivially have $hnr \,\Box\, m_\dagger \,\Box\, R_A\ m$. Intuitively, we want to compose these two refinements, to obtain $hnr \,\Box\, m_\dagger \,\Box\, (R_A \circ R_R)\ m'$. However, as our definition of *hnr* does not form a supremum, this would require $\$_a + \$_b \leq \$_a$ or $\$_a + \$_b \leq \$_b$, which obviously does not hold.

We have not yet found a way to define *hnr* or $\Downarrow_D$ in a form that does not exhibit this effect. Instead, we explicitly require that the supremum of the data refinement has a witness. The predicate *attains_sup* $m\ m'\ R_R$ characterizes that situation: it holds, if for all results $r$ of $m$ the supremum of the set of all abstractions $(r,r')\in R_R$ applied to $m'$ is in that set. This trivially holds if $R_R$ is *single-valued*, i.e. any concrete value is related with at most one abstract value, or if $m'$ is *one-time*, i.e. assigns the same resource bound to all its results.

In practice we *do* encounter non-single-valued relations[14], but they only occur as intermediate results where the composition with an *hnr* predicate is not necessary. Also, collapsing synthesis predicates and refinements in the NREST-monad typically is performed for the final algorithm whose running time does not depend on the result, thus is *one-time*, and ultimately *attains_sup*.

### 5 Case Study: Introsort

In this section, we apply our framework to the introsort algorithm [22]. We build upon the verification of its functional correctness [17] to verify its running time analysis and synthesize competitive efficient LLVM code for it. Following the "top-down" mantra, we use several intermediate steps to refine a specification down to an implementation.

#### 5.1 Specification of Sorting

We start with the specification of sorting a slice of a list:

$slice\_sort_{spec}\ xs_0\ l\ h\ (T) =$
  `assert` $(l{\leq}h \wedge h{\leq}length\ xs_0)$;
  `spec` $(\lambda xs.\ slice\_sort\_aux\ xs_0\ l\ h\ xs)\ (\lambda\_.\ T)$

where *slice_sort_aux* $xs_0\ l\ h\ xs$ states that $xs$ is a permutation of $xs_0$, $xs$ is sorted between $l$ and $h$ and equal to $xs_0$ anywhere else.

#### 5.2 Introsort's Idea

The introsort algorithm is based on quicksort. Like quicksort, it finds a pivot element, partitions the list around the pivot, and recursively sorts the two partitions. Unlike quicksort, however, it keeps track of the recursion depth, and if it

[14] The relation *oarr*, described in earlier work [17, 4.2] by one of the authors, is used to model ownership of parts of a list on an abstract level and is an example for a relation that is not single-valued.

exceeds a certain value (typically $\lfloor 2\log n \rfloor$), it falls back to heapsort to sort the current partition. Intuitively, quicksort's worst-case behaviour can only occur when unbalanced partitioning causes a high recursion depth, and the introsort algorithm limits the recursion depth, falling back to the $O(n \log n)$ heapsort algorithm. This combines the good practical performance of quicksort with the good worst-case complexity of heapsort.

Our implementation of introsort follows the implementation of *libstdc++*, which includes a second optimization: a first phase executes quicksort (with fallback to heapsort), but stops the recursion when the partition size falls below a certain threshold $\tau$. Then, a second phase sorts the whole list with one final pass of insertion sort. This exploits the fact that insertion sort is actually faster than quicksort for *almost-sorted* lists, i.e., lists where any element is less than $\tau$ positions away from its final position in the sorted list. While the optimal threshold $\tau$ needs to be determined empirically, it does not influence the worst-case complexity of the final insertion sort, which is $O(\tau n) = O(n)$ for constant $\tau$. The threshold $\tau$ will be an implicit parameter from now on.

While this seems like a quite concrete optimization, the two phases are already visible in the abstract algorithm, which is defined as follows in NREST:

$$
\begin{array}{ll}
\textit{introsort xs l h} = & \\
\quad \texttt{assert}(l \le h); & \\
\quad n \leftarrow \texttt{return}\ h{-}l; & (\$_{sub}) \\
\quad \texttt{if}_c\ n > 1\ \texttt{then} & (\$_{lt}) \\
\quad\quad xs \leftarrow \textit{almost\_sort}_{spec}\ xs\ l\ h; & (\$_{almost\_sort}) \\
\quad\quad xs \leftarrow \textit{final\_sort}_{spec}\ xs\ l\ h & (\$_{final\_sort}) \\
\quad\quad \texttt{return}\ xs & \\
\quad \texttt{else return}\ xs &
\end{array}
$$

where $\textit{almost\_sort}_{spec}$ $(T)$ specifies an algorithm that almost-sorts a list, consuming at most $T$ resources and $\textit{final\_sort}_{spec}$ $(T)$ specifies an algorithm that sorts an almost-sorted list, consuming at most $T$ resources.

The program *introsort* leaves trivial lists unchanged and otherwise executes the first and second phase. Its resource usage is bounded by the sum of the first and second phase and some overhead for the subtraction, comparison, and *if-then-else*. Using the verification condition generator we prove that *introsort* is correct, i.e., refines the specification of sorting a slice:

$$\textit{introsort xs l h} \le \Downarrow_C E_{is}\ (\textit{slice\_sort}_{spec}\ xs\ l\ h\ (\$_{sort}))$$

where $E_{is} = 0(\textit{sort}{:=}\textit{introsort}_{cost})$ is the exchange rate used at this step and $\textit{introsort}_{cost} = \$_{sub} + \$_{if} + \$_{lt} + \$_{almost\_sort} + \$_{final\_sort}$ is the total allotted cost for introsort.

#### 5.3 Quicksort Scheme

The first phase can be implemented in the following way:

$\textit{introsort\_aux}\ \mu\ xs\ l\ h =$

```
2      d ← depth_spec l h;                         ($_depth)
3      rec_c (λintrosort_rec (xs,l,h,d).
4        assert (l ≤ h);
5        n ← h−l;                                  ($_sub)
6        if_c n > τ then                           ($_lt)
7          if_c d = 0 then                         ($_eq)
8            slice_sort_spec xs l h                ($_sort_c (μ (h-l)))
9          else
10           (xs,m) ← partition_spec xs l h;       ($_partition_c (h-l))
11           d' ← d − 1;                           ($_sub)
12           xs ← introsort_rec (xs,l,m,d');
13           xs ← introsort_rec (xs,m,h,d');
14           return xs
15       else return xs
16     ) (xs,l,h,d)
```

where $partition_{spec}$ partitions a slice into two non-empty partitions, returning the start index $m$ of the second partition, and $depth_{spec}$ specifies $\lfloor 2\log(h-l) \rfloor$.

Let us first analyze the recursive part: if the slice is shorter than the threshold $\tau$, it is simply returned (line 15). Unless the recursion depth limit is reached, the slice is partitioned using $h - l$ $partition_c$ coins, and the procedure is called recursively for both partitions (lines 10-14). Otherwise, the slice is sorted at a price of $\mu\ (h{-}l)$ $sort_c$ coins (line 8). The function $\mu$ here represents the leading term in the asymptotic costs of the used sorting algorithm, and the $sort_c$ coin can be seen as the constant factor. This currency will later be exchanged into the respective currencies that are used by the sorting algorithm. Note that we use currency $sort_c$ to describe costs per comparison of a sorting algorithm, while currency *sort* describes the cost for a whole sorting algorithm.

Showing that the procedure results in an almost-sorted list is straightforward. The running time analysis, however, is a bit more involved. We presume a function $\mu$ that maps the length of a slice to an upper bound on the abstract steps required for sorting the slice. We will later use heapsort with $\mu_{nlogn}\ n = n \log n$.

Consider the recursion tree of a call in *introsort_rec*: We pessimistically assume that for every leaf in the recursion tree we need to call the fallback sorting algorithm. Furthermore, we have to partition at every inner node. This has cost linear in the length of the current slice. For each following inner level the lengths of the slices add up to the current one's, and so do the incurred costs. Finally we have some overhead at every level including the final one. The cost of the recursive part of *introsort_aux* is:

$$introsort\_rec_{cost}\ \mu\ (n,d) = \$_{sort_c}\ (\mu\ n) + \$_{partition_c}\ d * n \\ + ((d{+}1){*}n){*}(\$_{if}\ 2 + \$_{call}\ 2 + \$_{eq} + \$_{lt} + \$_{sub}\ 2)$$

The correctness of the running time bound is proved by induction over the recursion of *introsort_rec*. If the recursion limit is reached ($d{=}0$), the first summand pays for the fallback sorting algorithm. If $d{>}0$, part of the second summand pays for the partitioning of the current slice, then the list is split into two and the recursive costs are payed for by parts of all three summands. To bound the costs for the fallback sorting algorithm, $\mu$ needs to be *superadditive*: $\mu\ a + \mu\ b \leq \mu\ (a{+}b)$. In both cases, the third summand pays for the overhead in the current call.

For $d{=}\lfloor 2\log n \rfloor$ and an $O(n \log n)$ fallback sorting algorithm ($\mu{=}\mu_{nlogn}$), $\mathit{introsort\_rec}_{cost}\ \mu_{nlogn}$ is in $O(n \log n)$.[15] In fact, any $d{\in}O(\log n)$ would do.

Before executing the recursive method, *introsort_aux* calculates the depth limit $d$. The correctness theorem then reads:

$$\mathit{introsort\_aux}\ \mu_{nlogn}\ xs\ l\ h \leq \Downarrow_C (E_{isa} (h{-}l)) (\mathit{almost\_sort}_{spec}\ xs\ l\ h\ \$_{almost\_sort})$$

with $E_{isa}\ n = 0(\mathit{almost\_sort}{:=}\ \$_{depth} + \mathit{introsort\_rec}_{cost}\ \mu_{nlogn}\ (n,\ \lfloor 2\log n \rfloor))$.

Note that specifications typically use a single coin of a specific currency for their abstract operation, which is then exchanged for the actual costs, usually depending on the parameters.

This concludes the interesting part of the running time analysis of the first phase. It is now left to plug in an $O(n \log n)$ fallback sorting algorithm, and a linear partitioning algorithm.

*Heapsort* Independently of introsort, we have proved correctness and worst-case complexity of heapsort, yielding the following refinement lemma:

$$\mathit{heapsort}\ xs\ l\ h \leq \Downarrow_C (E_{hs}\ (h{-}l))\ (\mathit{slice\_sort}_{spec}\ xs\ l\ h\ (\$_{sort}))$$

where $E_{hs}\ n = 0(\mathit{sort}{:=}\ c_1 + \mathit{log}\ n * c_2 + n * c_3 + (n * \mathit{log}\ n) * c_4)$ for some constants $c_i :: \mathit{ecost}$.

Assuming that $n \geq 2$,[16] we can estimate $E_{hs}\ n\ \mathit{sort} \leq \mu_{nlogn}\ n * c$, for $c = c_1 + c_2 + c_3 + c_4$, and thus get, for $E_{hs'} = 0(\mathit{sort}_c := c)$:

$$\begin{aligned}&\Downarrow_C (E_{hs}\ (h{-}l))\ (\mathit{slice\_sort}_{spec}\ xs\ l\ h\ (\$_{sort}))\\ &\leq \Downarrow_C E_{hs'}\ (\mathit{slice\_sort}_{spec}\ xs\ l\ h\ (\$_{sort_c}\ (\mu_{nlogn}\ (h{-}l))))\end{aligned}$$

and, by, transitivity

$$\mathit{heapsort}\ xs\ l\ h \leq \Downarrow_C E_{hs'}\ (\mathit{slice\_sort}_{spec}\ xs\ l\ h\ (\$_{sort_c}\ (\mu_{nlogn}\ (h{-}l))))$$

Note that our framework allowed us to easily convert the abstract currency from a single operation-specific *sort* coin to a $\mathit{sort}_c$ coin for each comparison operation.

*Partition and Depth Computation* We implement partitioning with the Hoare partitioning scheme using the median-of-3 as the pivot element. Moreover, we implement the computation of the depth limit ($2\lfloor \log(h - l) \rfloor$) by a loop that counts how often we can divide by two until zero is reached. This yields the following refinement lemmas:

$$\begin{aligned}&\mathit{pivot\_partition}\ xs\ l\ h \leq \Downarrow_C E_{pp}\ (\mathit{partition}_{spec}\ xs\ l\ h\ (\$_{partition_c}\ (h{-}l)))\\ &\mathit{calc\_depth}\ l\ h \leq \Downarrow_C (E_{cd}\ (h{-}l))\ (\mathit{depth}_{spec}\ l\ h\ (\$_{depth}))\end{aligned}$$

[15] More precisely, the sum over all (finitely many) currencies is in $O(n \log n)$.

[16] Note that this is a valid assumption, as heapsort will never be called for trivial slices.

*Combining the Refinements* We replace $\mathit{slice\_sort}_{spec}$, $\mathit{partition}_{spec}$ and $\mathit{depth}_{spec}$ by their implementations *heapsort*, *pivot_partition* and *calc_depth*. We call the resulting implementation $\mathit{introsort\_aux}_2$, and prove

$$\mathit{introsort\_aux}_2\ xs\ l\ h \leq \Downarrow_C (E_{aux}\ (h{-}l))\ (\mathit{introsort\_aux}\ \mu_{nlogn}\ xs\ l\ h)$$

where the exchange rate $E_{aux}$ combines the exchange rates $E_{hs'}$, $E_{pp}$ and $E_{cd}$ for the component refinements.

Transitive combination with the correctness lemma for *introsort_aux* then yields the correctness lemma for $\mathit{introsort\_aux}_2$:

$$\mathit{introsort\_aux}_2\ xs\ l\ h \leq \Downarrow_C (E_{isa2}\ (h{-}l))\ (\mathit{almost\_sort}_{spec}\ xs\ l\ h\ (\$_{almost\_sort}))$$

where $E_{isa2}\ n = 0(\mathit{almost\_sort}{:=}\downarrow_C (E_{aux}\ n)\ (\mathit{introsort\_aux}_{cost}\ n))$ and the operation $\downarrow_C E\ t$ applies an exchange rate to a resource function.

*Refining Resources* The stepwise refinement approach allows to structure an algorithm verification in a way that correctness arguments can be conducted on a high level and implementation details can be added later. Resource currencies permit the same for the resource analysis of algorithms: they summarize compound costs, allow reasoning on a higher level of abstraction and can later be refined into fine-grained costs. For example, in the resource analysis of *introsort_aux* the currencies $\mathit{sort}_c$ and $\mathit{partition}_c$ abstract the cost of the respective subroutines. The abstract resource argument is independent from their implementation details, which are only added in a subsequent refinement step, via the exchange rate $E_{aux}$.

#### 5.4 Final Insertion Sort

The second phase is implemented by insertion sort, repeatedly calling the subroutine *insert*. The specification of *insert* for an index $i$ captures the intuition that it goes from a slice that is sorted up to index $i{-}1$ to one that is sorted up to index $i$. Insertion is implemented by moving the last element to the left, as long as the element left of it is greater (or the start of the list has been reached). Moving an element to its correct position takes at most $\tau$ steps, as after the first phase the list is almost sorted, i.e., any element is less than $\tau$ positions away from its final position in the sorted list. Moreover, elements originally at positions greater $\tau$ will never reach the beginning of the list, which allows for the *unguarded* optimization. It omits the bounds check for those elements, saving one index comparison in the innermost loop. Formalizing these arguments yields the implementation *final_insertion_sort* that satisfies

$$\mathit{final\_insertion\_sort}\ xs\ l\ h \leq \Downarrow_C (E_{fis}(h{-}l))\ (\mathit{final\_sort}_{spec}\ xs\ l\ h\ (\$_{final\_sort}))$$

where $E_{fis}\ n = 0(\mathit{final\_sort}{:=}\mathit{final\_insertion}_{cost}\ n)$, and $\mathit{final\_insertion}_{cost}\ n$ is linear in $n$.

Note that *final_insertion_sort* and $\mathit{introsort\_aux}_2$ use the same currency system. Plugging both refinements into *introsort* yields $\mathit{introsort}_2$ and the lemma

$$\mathit{introsort}_2\ xs\ l\ h \leq \Downarrow_C (E_{is2}(h{-}l))\ (\mathit{introsort}\ xs\ l\ h)$$

where the exchange rate $E_{is2}$ combines the rates $E_{isa2}$ and $E_{fis}$.

#### 5.5 Separating Correctness and Complexity Proofs

A crucial function in heapsort is *sift_down*, which restores the heap property by moving the top element down in the heap. To implement this function, we first prove correct a version $\mathit{sift\_down}_1$, which uses swap operations to move the element. In a next step, we refine this to $\mathit{sift\_down}_2$, which saves the top element, then executes upward moves instead of swaps, and, after the last step, moves the saved top element to its final position. This optimization spares half of the memory accesses, exploiting the fact that the next swap operation will overwrite an element just written by the previous swap operation.

However, this refinement is not structural: it replaces swap operations by move operations, and adds an additional move operation at the end. At this point, we chose to separate the functional correctness and resource aspect, to avoid the complexity of a combined non-structural functional and currency refinement. It turns out that proving the complexity of the optimized version $\mathit{sift\_down}_2$ directly is straightforward. Thus, as sketched in Section 2.4, we first prove[17] $\mathit{sift\_down}_2 \le \mathit{sift\_down}_1 \le \mathit{sift\_down}_{spec}\ (\infty)$, ignoring the resource aspect. Separately, we prove $\mathit{sift\_down}_2 \le_n \mathtt{spec}\ (\lambda\_.\ \mathit{True})\ \mathit{sift\_down}_{cost}$, and combine the two statements to get $\mathit{sift\_down}_2 \le \mathit{sift\_down}_{spec}\ \mathit{sift\_down}_{cost}$.

#### 5.6 Refining to LLVM

The above abstract programs implicitly come with a fixed type and comparison operator for the elements of the list to be sorted. Those programs use abstract operations and currencies for arithmetic operations on indexes, control flow, comparisons and read/write of a random-access iterator (abstracted by lists with update and lookup operations).

When we further assume an LLVM program that refines the comparison operator in LLVM, and specify how the random-access data structure should be implemented — we choose arrays — we can automatically synthesize an LLVM program *introsort_impl* that refines $\mathit{introsort}_2$, i.e., satisfies the theorem:

$$\begin{array}{l} \mathit{hnr}\ (\mathit{array}_A\ p\ \mathit{xs} \star \mathit{snat}_A\ l_\dagger\ l \star \mathit{snat}_A\ h_\dagger\ h) \\ \quad (\mathit{introsort\_impl}\ p\ l_\dagger\ h_\dagger) \\ \quad (\mathit{snat}_A\ l_\dagger\ l \star \mathit{snat}_A\ h_\dagger\ h)\ \mathit{array}_A\ (\mathit{introsort}_2\ \mathit{xs}\ l\ h) \end{array}$$

Combination with the refinement lemmas for $\mathit{introsort}_2$ and *introsort*, followed by conversion to a Hoare triple, yields our final correctness statement:

$$\begin{array}{l} l \le h \wedge h < \mathit{length}\ \mathit{xs}_0 \implies \\ \{\$(\mathit{introsort\_impl}_{cost}\ (h-l)) \star \mathit{array}_A\ p\ \mathit{xs}_0 \star \mathit{snat}_A\ l_\dagger\ l \star \mathit{snat}_A\ h_\dagger\ h\} \\ \quad \mathit{introsort\_impl}\ p\ l_\dagger\ h_\dagger \\ \{\lambda r.\ \exists_A \mathit{xs}.\ \mathit{array}_A\ r\ \mathit{xs} \star \uparrow(\mathit{slice\_sort\_aux}\ \mathit{xs}_0\ l\ h\ \mathit{xs}) \star \mathit{snat}_A\ l_\dagger\ l \star \mathit{snat}_A\ h_\dagger\ h\} \end{array}$$

where $\mathit{introsort\_impl}_{cost} :: \mathit{nat} \rightarrow \mathit{ecost}$ is the cost bound obtained from applying the exchange rates $E_{is}$ and then $E_{is2}$ to $\$_{sort}$.

[17] Note that we have omitted the function parameters for better readability.

Note that this statement is independent of the Refinement Framework. Thus, to believe in its meaningfulness, one has to only check the formalization of Hoare triples, separation logic, and the LLVM semantics.

To formally prove the statement "*introsort_impl has complexity* $O(n \log n)$", we observe that $introsort\_impl_{cost}$ uses only finitely many currencies, and only finitely many coins of each currency. We define the overall number of coins as

$$introsort\_impl_{allcost}\ n = \Sigma c.\ introsort\_impl_{cost}\ n\ c$$

which expands to

$$introsort\_impl_{allcost}\ n = 4693 + 5 * \ log\ n + 231 * n + 455 * (n * log\ n)$$

which, in turn, is routinely proved to be in $O(n \log n)$.

As a last step, we instantiate the element type to 64-bit unsigned integers and the comparison operation to LLVM's *icmp_ult* instruction, to obtain a program that sorts integers in ascending order. Our code generator can export this to actual LLVM text and a corresponding header file for interfacing our sorting algorithm from C or C++.

As LLVM does not support generics, we cannot implement a replacement for C++'s generic *std::sort<T>*. However, by repeating the last step for different types and compare operators, we can implement a replacement for any fixed $T$.

#### 5.7 Benchmarks

In this section we present benchmarks comparing the code extracted from our formalization with the real world implementation of introsort from the GNU C++ Library (*libstdc++*). Also, as a regression test, we compare with the code extracted from an earlier formalization of introsort [17] that did not verify the running time complexity and used an earlier iteration of the Sepref framework and LLVM semantics without time.

The results are shown in Figure 1. As expected, all three implementations have similar running times. Note that the small differences are well within the noise of the measurements. We conclude that adding the complexity proof to our introsort formalization, and the time aspect to our refinement process has not introduced any timing regressions in the generated code. Note, however, that the code generated by our current formalization is not identical to what the original formalization generated. This is mainly due to small changes in the formalization introduced when adding the timing aspect.

### 6 Conclusions

We have presented a refinement framework for the simultaneous verification of functional correctness and complexity of algorithm implementations with competitive practical performance.

We use stepwise refinement to separate high-level algorithmic ideas from low-level optimizations, enabling convenient verification of highly optimized algorithms. The novel concept of resource currencies also allows structuring of the

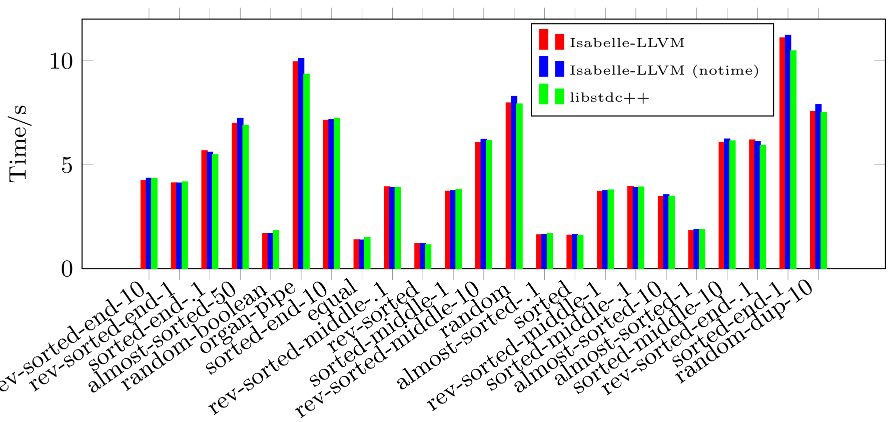

**Fig. 1.** Comparison of the running time measured for the code generated by the formalization described in this paper (Isabelle-LLVM), the original formalization from [17] (notime), and the *libstdc++* implementation. Arrays with $10^8$ *uint64*s with various distributions were sorted, and we display the smallest time of 10 runs. The programs were compiled with *clang-10 -O3*, and run on an Intel XEON E5-2699 with 128GiB RAM and 256K/55M L2/L3 cache. See [17] for details of the benchmarking method.

complexity proofs along the refinement chain. Our framework refines down to the LLVM intermediate representation, such that we can use a state-of-the-art compiler to generate performant programs.

As a case-study, we have proved the functional correctness and complexity of the introsort sorting algorithm. Our verified implementation performs on par with the (unverified) state-of-the-art implementation from the GNU C++ Library. It also provably meets the C++11 standard library [7] specification for *std::sort*, which in particular requires a worst-case time complexity of $O(n \log n)$. We are not aware of any other verified real-world implementations of sorting algorithms that come with a complexity analysis.

Our work is a combination and substantial extension of an earlier refinement framework for functional correctness [15] which also comes with a verification of introsort [17], and a refinement framework for a single *enat*-valued currency [11]. In particular, we have generalized the refinement framework to arbitrary resources, introduced currencies that help organizing refinement proofs, extended the LLVM semantics and reasoning infrastructure with a cost model, connected it to the refinement framework via a new version of the Sepref tool, and, finally, added the complexity analysis for introsort.

#### 6.1 Related Work

Nipkow *et al.* [23, §4.1] collect verification efforts concerning sorting algorithms. We add a few instances verifying running time: Wang *et al.* use TiML [25] to verify correctness and asymptotic time complexity of mergesort automatically.

Zhan and Haslbeck [26] verify functional correctness and asymptotic running time analysis of imperative versions of insertion sort and mergesort. We build on earlier work by Lammich [17] and provide the first verification of functional correctness and asymptotic running time analysis of heapsort and introsort.

The idea to generalize the nres monad [19] to resource types originates from Carbonneaux *et al.* [4]. They use potential functions ($state \to enat$) instead of predicates ($state \to bool$), present a quantitative Hoare logic and extend the CompCert compiler to preserve properties of stack-usage from programs in Clight to compiled programs.

We see our paper in the line of research concerning simultaneously verifying functional correctness and worst-case time complexity of algorithms. Atkey [1] pioneered resource analysis with separation logic, Guéneau *et al.* [9] present a framework that uses time credits in Coq and apply it to involved algorithms and data structures [10,6]. We further develop their work in three ways: First, while time credits usually are natural numbers [1,9,26,21,6] or integers [10], we generalize to an abstract resource type and specifically use resource currencies for a fine-grained analysis. Second, we use stepwise refinement to structure the verification and make the resource analysis of larger use-cases manageable. Third, we provide facilities to automatically extract efficient competitive code from the verification. The following are the most complex algorithms and data structures with verified running time analysis using time credits and separation logic we are aware of: a linear time selection algorithm [26], an incremental cycle detection algorithm [10], Union-Find [6], Edmonds-Karp and Kruskal's algorithm [11].

#### 6.2 Future Work

A verified compiler down to machine code would further reduce the trusted code base of our approach. While that is not expected to be available soon for LLVM in Isabelle, the NREST-monad and the Sepref tool are general enough to connect to a different back end. Formalizing one of the CompCert C semantics [2] in Isabelle, connecting it to the NREST-monad and then processing synthesized C code with CompCert's verified compiler would be a way to go.

In this paper we apply our framework to verify an involved algorithm that only uses basic data structures, i.e. arrays. A next step is to verify more involved data structures, e.g. by porting existing verifications of the Imperative Collections Framework [16] to LLVM. We do not yet see how to reason about the running time of data structures like hash maps, where worst-case analysis would be possible but not useful. In general, extending the framework to average-case analysis and probabilistic programs are exciting roads to take.

We plan to implement more automation, saving the user from writing boilerplate code when handling resource currencies and exchange rates.

Neither the LLVM nor the NREST level of our framework is tied to running time. Applying it to other resources like maximum heap space consumption might be a next step.
